# An Unusual Consolidation: Lobar Pulmonary Hemorrhage Due to Antithrombotic Therapy

**DOI:** 10.5811/cpcem.2017.7.34462

**Published:** 2017-10-06

**Authors:** Katrina D’Amore, David Traficante, Terrance McGovern, Marco Propersi, Stacey Barnes

**Affiliations:** St. Joseph’s Regional Medical Center, Department of Emergency Medicine, Paterson, New Jersey

## Abstract

Alveolar hemorrhage is a rare yet devastating clinical entity if not identified and treated aggressively. Exceedingly rare are the cases of anticoagulant-induced alveolar hemorrhage with very few cases described in the current literature. The nonspecific presentation of an alveolar hemorrhage makes its diagnosis and appropriate treatment difficult in the emergency department. We report a case of a patient on warfarin for atrial fibrillation who was initially misdiagnosed as having community-acquired pneumonia, but subsequently was identified to have a fatal alveolar hemorrhage.

## INTRODUCTION

Pulmonary hemorrhage can have numerous potential etiologies such as pulmonary embolism, Wegener’s granulomatosis, Goodpasture syndrome, systemic lupus erythematosus and many drugs, including warfarin.^1^ To the best of our knowledge the incidence of pulmonary hemorrhage induced by anticoagulant use has not been elucidated; however, with very few cases reported we suspect it is a rare diagnosis that may be becoming more common with increasing use of anticoagulants.^1^–^4^ The hallmark of treatment is to address the coagulopathy and provide supportive care with suitable oxygenation and ventilation if needed.^1^

## CASE REPORT

A 62-year-old male presented to the emergency department (ED) via Advanced Life Support with a two-day history of shortness of breath and subjective fevers. His past medical history was significant for congestive heart failure requiring an automatic implantable cardioverter-defibrillator, atrial fibrillation and chronic obstructive pulmonary disease. During the initial assessment, the patient was found to be afebrile, tachycardic (heart rate: 117 beats per minute, paced rhythm) and normotensive (blood pressure:116/66 mmHg); however, the patient was moderately dyspneic and hypoxic, requiring support with bilevel positive airway pressure ventilation. On chest auscultation the patient was found to have rhonchi in the left lower lobe and he was mildly tachypneic with a respiratory rate of 22 breaths per minute; otherwise, his physical examination was unremarkable.

**Image 1:**
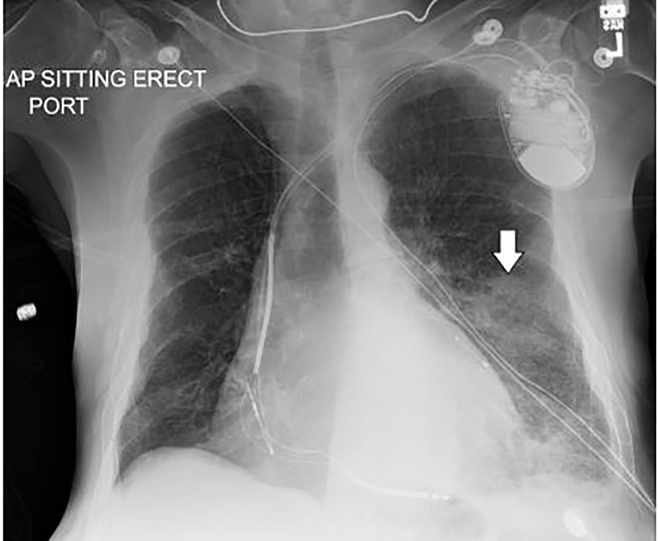
The initial anterior-posterior chest radiograph demonstrating a left lower lobe infiltrate (arrow).

**Image 2:**
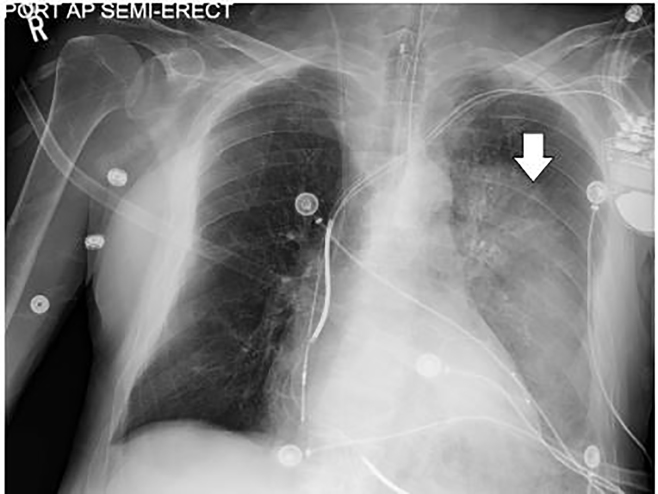
The follow-up anterior-posterior chest radiograph following the patient’s clinical decompensation demonstrating a rapidly worsening left lower lobe infiltrate (arrow).

The ED laboratory evaluation revealed a leukocytosis of 21.4 K/mm^3^ with bandemia of 15% and presumed left lower lobe infiltrate seen on chest radiograph (Image 1). The patient was also acutely anemic with hemoglobin of 12 g/dL as compared to his baseline of 14.4 g/dL three months earlier. The patient was initially managed with intravenous (IV) ceftriaxone and azithromycin for community-acquired pneumonia and admitted to the intensive care unit for close monitoring of his cardiopulmonary status. After an episode of massive hemoptysis and desaturation to 84%, the patient required endotracheal intubation for airway protection. His warfarin-induced coagulopathy (prothrombin time: 33.7 seconds; international normalized ratio: 3.3) was reversed with two units of fresh frozen plasma and IV Vitamin K. Repeat chest radiograph (Image 2) two hours after the initial chest radiograph (Image 1) revealed interval worsening of the left lower lobe opacity confirmed as localized alveolar hemorrhage on bronchoscopy. Bronchial washings demonstrated hemosiderin-laden macrophages, while malignant cells were notably absent. Sputum cultures obtained after seven days post-intubation grew *P. aeruginosa*. The patient subsequently succumbed to his illness.

## DISCUSSION

Pulmonary hemorrhage is a disease process classically characterized by hemoptysis, anemia, and pulmonary opacities on chest radiography; however, hemoptysis is initially absent in up to one-third of cases.^5^ Pulmonary hemorrhage can be further classified as diffuse alveolar or localized hemorrhage. While each of these sub-types can have a myriad of causes ranging from autoimmune and infectious to malignant etiology, both can arise as complications of medications.^5^ As in this case, anticoagulant therapies can be identified as a precipitating factor; however, a variety of medications such as amiodarone, nitrofurantoin, phenytoin, propylthiouracil, and abciximab are also commonly associated with pulmonary hemorrhage.^6^ Resultant mortality varies and is dependent on the underlying cause and comorbidities such as heart failure and renal disease. The presence of thrombocytopenia or sepsis is correlated with decreased survival.^7^ Treatment is aimed at addressing the underlying cause, prompt reversal of any anticoagulants, and providing supportive care, often in the form of invasive or non-invasive mechanical ventilation.

**CPC-EM Capsule**

What do we already know about this clinical entity?

Pulmonary hemorrhage is a rare entity resulting from a myriad of causes and associated with high mortality. The classic presentation is a triad of hemoptysis, anemia and opacities on chest radiograph.

What makes this presentation of disease reportable?

This case illustrates the difficulties in distinguishing pulmonary hemorrhage from other etiologies such as infectious consolidations and cardiogenic pulmonary edema on routine imaging.

What is the major learning point?

Emergency physicians should be aware of this diagnosis as it can be rapidly fatal, and effective treatment must be initiated quickly to target the underlying etiology.

How might this improve emergency medicine practice?

Increased awareness of rare but life-threatening diagnoses can increase detection and improve patient outcomes.

## CONCLUSION

Regardless of type or location, alveolar hemorrhage can be difficult to differentiate from infectious or cardiogenic etiologies on chest radiographs, making accurate diagnosis difficult in the ED.^6^ The opacities due to alveolar hemorrhage resolve slowly over several days and typically spare the costophrenic angles, the presence of which provide clues to the diagnosis.^6^ High resolution computed tomography will acutely demonstrate ground-glass attenuation, which can also characterize several other pulmonary conditions.^8^ Often, bronchoalveolar lavage is necessary and will show persistent or increasing blood return from repeated lavages. In the case presented here, the patient’s disproportional dyspnea compared to his initial radiographic findings and subsequent rapid respiratory failure may have been clues to an underlying pathology beyond community-acquired pneumonia. Differentiating the etiology of both localized or diffuse opacities on chest radiographs can be a diagnostic challenge, but can greatly influence the subsequent management and outcome for the patient.^9^
